# Solid-State Fermentation Drives L-Carnitine Accumulation and Isoflavone Bioconversion with Functional Enhancement in Soybean

**DOI:** 10.4014/jmb.2606.06042

**Published:** 2026-08-26

**Authors:** Tri Nhut Pham, Hayeong Kim, Ghahyun Jeffrey Kim, Soyoung Park, Taeyoung Um, Soochul Park, Doman Kim

**Affiliations:** 1 Graduate School of International Agricultural Technology, Seoul National University, Gangwon-do 25354, Republic of Korea; 2 Institute of Applied Technology and Sustainable Development, Nguyen Tat Thanh University, Ho Chi Minh City 700000, Vietnam; 3 Institute of Food Industrialization, Institutes of Green Bioscience and Technology, Seoul National University, Gangwon-do 25354, Republic of Korea; 4 Department of Clinical Pharmacy Practice, School of Pharmacy and Pharmaceutical Sciences, UC Irvine, CA 92617, USA; 5 Metabolic Engineering Division, National Institute of Agricultural Sciences, Rural Development Administration, Jeonju 54874, Republic of Korea; 6 Department of Plant Science, Kangwon National University, Gangneung 25457, Republic of Korea; 7 Mongta Incorporation, Digital-ro, Guro-gu, Seoul 08380, Republic of Korea

**Keywords:** Soybean, Solid-state fermentation, *Rhizopus oligosporus*, Isoflavones aglycone, Antioxidant activity, L-carnitine

## Abstract

Solid-state fermentation (SSF) is widely used to enhance the functional properties of plant-based substrates. In this study, soybean powder was used as a substrate for SSF with *Rhizopus oligosporus* for up to 20 days. SSF altered the physical properties of soybean, increasing its water-holding capacity by 1.4-fold, water-swelling capacity by 1.64-fold, oil-absorption capacity by 1.55-fold, and cholesterol-binding capacity by 1.49-fold at pH 2.0. Biochemical characterization of fermented soybean showed a 3.13-fold higher TPC, a 3.84-fold higher TFC, and a 2.52-fold higher TSC. L-carnitine content in soybean rose from 0.66 ± 0.15 mg/kg DM to 161.21 ± 3.78 mg/kg DM, representing a 244.26-fold increase over nonfermented soybean. Additionally, the isoflavone glucosides daidzin, genistin, and glycitin progressively decreased during fermentation and became undetectable by days 15–20, whereas their corresponding aglycones exhibited compound-specific and time-dependent changes. Daidzein rose from 37.26 ± 19.16 μmol/kg DM to 691.06 ± 36.98 μmol/kg DM after 15 days. Genistein, initially undetectable, reached 1212.44 ± 43.32 μmol/kg DM after 10 days. Meanwhile, glycitein increased from an initial 82.96 ± 2.48 μmol/kg DM to 238.70 ± 4.91 μmol/kg DM. The soybean's antioxidant activity, measured by ORAC, FRAP, and ABTS assays, increased by 1.93-, 2.93-, and 1.5-fold, respectively. Its inhibitory effects on α-glucosidase and mushroom tyrosinase improved by 2.31- and 1.5-fold, respectively. These results indicate that fermented soybean has potential as a functional ingredient in the nutraceutical, cosmeceutical, and pharmaceutical industries.

## Introduction

Soybean (*Glycine max*) is a nutritionally dense legume containing high levels of protein, fiber, plant-based fat, vitamins, and diverse secondary metabolites, including isoflavones, saponins, peptides, and phenolic compounds [1-4]. Soybean products are popular around the world due to their health benefits, such as antioxidant, anti-diabetic, anti-inflammatory, and cardiovascular-protective effects [5]. However, its broader acceptance faces several challenges due to compositional and sensory concerns. These include enzyme inhibitors, such as proteinase and trypsin inhibitors, that reduce digestibility. Moreover, soy oligosaccharides such as raffinose and stachyose serve as antinutritional factors, while the characteristic beany or fishy flavors can also lower consumer appeal [6]. Fermentation has emerged as a practical, eco-friendly technique to enhance the nutritional, biochemical, and sensory profiles of soybeans. This process elevates bioactive compounds, drives enzymatic bioconversion, and minimizes undesirable components [7-9]. Solid-state fermentation (SSF) is an economically viable method that not only enriches the nutritional value of food materials but also generates key secondary metabolites, including pigments, organic acids, phenolics, vitamins, and flavors, while effectively removing antinutritional factors during fermentation [5, 10]. Tempeh is a traditional functional food produced from soybeans via fermentation with *Rhizopus* spp., originating in Indonesia. SSF for tempeh production is gaining global traction as a cost-effective, sustainable, and health-promoting technology for legume-based food development. During fermentation, *Rhizopus* strains produce enzymes, including proteases, lipases, carbohydrases, and phytases. These enzymes digest the substrate, degrade major antinutritional factors, and synthesize essential nutrients and bioactive compounds [11]. Zhang *et al*. (2022) discovered that fermenting whole soybeans with *R. oligosporus* RT-3 for 60 h resulted in a broad enzyme system, including α-amylase, endoglucanase, exoglucanase, β-glucosidase, elastase, and protease. This fermentation enhanced levels of small peptides, isoflavone aglycones, and antioxidant activity, as measured by FRAP and ABTS assays. Although most research on soybean fermentation focuses on whole soybeans, milling is increasingly recognized as a key step in creating functional materials [12]. In our previous study, maca powder fermented by *R. oligosporus* showed peak microbial growth on day 10. Meanwhile, L-carnitine, which facilitates the transport of long-chain fatty acids into mitochondria for β-oxidation, accumulated continuously, peaking after 15 days. Interestingly, the highest yields of total phenolics, total flavonoids, and macamide B were concurrently observed on day 10 [13]. Therefore, this study aims to explore how solid-state fermentation of soybean powder with *R. oligosporus* over a long period, up to 20 days affects its physical properties, such as water holding capacity (WHC), water swelling capacity (WSC), oil absorption capacity (OAC), and cholesterol binding capacity (CBC), as well as its phytochemical compounds, including total phenolics (TPC), total flavonoids (TFC), and total saponins (TSC). It also assesses bioactive compounds, including ergosterol, L-carnitine, and isoflavone glycosides, as well as their aglycones, during fermentation. Furthermore, we evaluated the functional properties of soybean, including antioxidant activities measured by oxygen radical absorbance capacity (ORAC), ferric reducing antioxidant power (FRAP), and ABTS radical-scavenging activity, as well as its inhibitory effects on α-glucosidase and tyrosinase enzymes. Our results suggest that SSF with *R. oligosporus* enhances the value of soybean powder as a functional ingredient for use in the nutraceutical, cosmeceutical, and pharmaceutical industries.

## Materials and Methods

### Materials

Soybean (*Glycine max*, Daewon variety) was provided by the National Institute of Agricultural Science, Rural Development Administration (Republic of Korea). L-carnitine, daidzein, genistein, daidzin, and genistin were sourced from Tokyo Chemical Industry Co., Ltd. (Japan), while glycitin, glycitein, and 2,2′-azobis(2-amidinopropane) dihydrochloride (AAPH) were supplied by ChemFaces (China). α-Glucosidase (from *Saccharomyces cerevisiae*), 6-hydroxy-2,5,7,8-tetramethylchroman-2-carboxylic acid (Trolox), ergosterol, p-nitrophenyl-β-D-glucopyranoside (p-NPG), L-3,4-dihydroxyphenylalanine (L-DOPA), formic acid, gallic acid, p-nitrophenyl-β-D-glucopyranoside (p-NPG), and dimethyl sulfoxide (DMSO) were purchased from SigmaAldrich (USA). Fluorescein sodium salt was purchased at Alfa Aesar (USA). All remaining solvents and reagents were of analytical grade.

### Solid-State Fermentation

*R. oligosporus* KCCM 11948P was acquired from a prior study and cultivated on potato dextrose agar (PDA; Difco, USA) at 30°C for 5 days to generate spores [14]. Soybean powder (5 g) was placed in a glass Petri dish (60 × 15 mm) and soaked in distilled water at a 1:1 (w/w) ratio for 12 h at 22°C. Water activity (a_w_) was measured before and after soaking using a water activity meter (AquaLab Series 3TE, USA), with a_w_ ≥ 0.98. The samples were then autoclaved (JSAT-105; JSR, Republic of Korea) at 121°C for 20 min and cooled to 22°C. A spore suspension containing 5 × 10^5^ spores/mL of *R. oligosporus* was inoculated onto the soybean, which was then incubated at 30°C for 3, 5, 7, 10, 15, and 20 days. The growth of *R. oligosporus* was regularly tracked by observing its appearance macroscopically and checking microscopically for contamination. Throughout fermentation, normal fungal growth was maintained, and only Petri dishes exhibiting growth without visible contamination were chosen for further analysis. After fermentation, the samples were frozen at -80°C and lyophilized at 0°C and 10 Pa using a freeze-dryer (Eyela FD-550; Rikakikai Co., Japan).

### Sample Extraction

For extraction, a 0.5 g sample was shaken with 10 mL of 80% (v/v) ethanol at 150 rpm and 30°C for 1 h. The extracts were sonicated (VCX 130 Ultrasonic Processor, Sonics and Materials, Inc., USA) for 3 min at 40% amplitude (20 W output) in pulse mode (5 cycles) on ice. The extracts were then centrifuged at 8,000 × g for 15 min at 4°C, and the supernatants were collected. The remaining pellets were re-extracted twice under the same conditions. The supernatants were pooled and filtered through Whatman No. 1 filter paper (USA). Ethanol in the filtrates was removed using an evaporator (Heidolph Instruments, Germany). The extracted samples were then freeze-dried at 0°C and 10 Pa before analysis.

### Physicochemical Characterization

**Water holding capacity (WHC) assay.** Nonfermented and fermented soybeans (50 mg) were combined with 1 mL of deionized water and hydrated at 22°C for 24 h. The mixture was then centrifuged at 10,000 × g for 15 min, after which the supernatant was discarded. The hydrated residue was collected and weighed. WHC was calculated as follows:

$$\text{WHC (g/g) =}\frac{{\text{W}}_{2}-{\text{W}}_{1}}{{\text{W}}_{1}}$$

where W_1_ is the initial dry sample weight (g), and W_2_ is the weight of the hydrated residue after centrifugation (g).

**Water swelling capacity (WSC) assay.** WSC was assessed by hydrating 50 mg of both nonfermented and fermented soybean powder in 1 mL of deionized water at 4°C for 24 h. After hydration, the swollen sample volume was measured, and WSC was calculated accordingly.

$$\text{WSC }\left(\text{mL/g}\right)=\frac{{\text{v}}_{2}-{\text{v}}_{1}}{{\text{W}}_{1}}$$

where v_1_ is the initial volume of the dry sample (mL), v_2_ is the volume of the hydrated sample (mL), and W_1_ is the dry sample weight (g).

**Oil absorption capacity (OAC) assay.** OAC was measured by combining 50 mg of fermented soybean powder with 1 mL of olive oil, followed by incubation at 22°C for 24 h. The mixture was centrifuged at 10,000 × g for 15 min, after which the supernatant was discarded. The residue, which absorbed the oil, was recovered and weighed. OAC was calculated accordingly:

$$\text{OAC}\left(\text{g/g}\right)\text{=}\frac{{\text{W}}_{\text{2}}{\text{-W}}_{\text{1}}}{{\text{W}}_{\text{1}}}$$

where W_1_ is the initial dry sample weight (g), and W_2_ is the weight of the oil-absorbed residue after centrifugation (g).

**Cholesterol binding capacity (CBC) assay.** A 500 mg sample of nonfermented or fermented soybean was combined with 5 mL of 10% egg yolk solution, with the pH adjusted to either 2.0 or 7.0. The mixture was incubated at 37°C with gentle shaking at 120 rpm for 2 h. It was then centrifuged at 4,000 × g for 20 min to obtain the supernatant. Next, 4 mL of 95% ethanol was added, and the mixture was centrifuged again at 4,000 × g for 20 min. The ethanol was evaporated at 40°C. Cholesterol levels were measured by reacting the sample with sulfuric acid, 0.05% (w/v) o-phthalaldehyde, and glacial acetic acid at a 1:5:1:1 (v/v/v/v) ratio, followed by incubation at 25°C for 20 min. Absorbance was measured at 550 nm using a SpectraMax M3 microplate reader (Molecular Devices, USA). Results are given as milligrams of cholesterol per gram of sample (mg/g).

$$\text{CBC}\left(\text{mg/g}\right)\text{=}\frac{{\text{C}}_{\text{y}}{\text{-C}}_{\text{s}}}{{\text{W}}_{\text{1}}\left(\text{g}\right)}\text{×DF}$$

where C_y_ is the cholesterol concentration in the diluted egg yolk (mg/mL), C_s_ is the cholesterol concentration in the sample supernatant (mg/mL), W_1_ is the sample weight (g), and DF is the dilution factor.

### Phytochemical Analysis

**Analysis of ergosterol.** Ergosterol analysis was conducted following our previous study [15], with minor modifications. Nonfermented and fermented soybean samples (300 mg) were saponified with 10 mL of 10% (w/v) KOH in methanol. The mixture was kept at 80°C for 60 min. After cooling to 22°C, 3 mL of distilled water and 4 mL of hexane were added and mixed thoroughly. The mixture was then allowed to phase-separate at 22°C, and the upper hexane layer, containing ergosterol, was collected. This extraction was repeated with an additional 4 mL of hexane. The collected hexane was evaporated under nitrogen, dissolved in methanol, and filtered through a 0.2 μm membrane syringe filter. Ergosterol content was quantified using a Waters UPLC system (Milford, USA) with a PDA detector set to 282 nm and a BEH C18 column (2.1 × 100 mm, 1.7 μm; Waters, USA). The mobile phase was isocratic acetonitrile at a flow rate of 0.3 mL/min for 10 min. Standard solutions of ergosterol were prepared in methanol at concentrations ranging from 0.1 to 60 μg/mL.

**Analysis of L-carnitine.** L-carnitine was analyzed using the method described in a previously published study [15]. Briefly, a 10 mg sample of the sample was dissolved in DMSO, then diluted with methanol to a final concentration of 1 mg/mL and filtered through a 0.2 μm syringe filter (Pall, USA). The analysis was performed using a Waters UPLC system with a BEH HILIC column (1.7 μm, 2.1 × 100 mm; Waters). Detection was performed using a QDa mass detector (Waters). The mobile phase consisted of acetonitrile with 0.1% formic acid (solvent A) and distilled water with 0.1% formic acid (solvent B). The gradient started at 70% A (0 min), increased to 80% at 1.0 min, to 95% at 3.0 min, and reached 100% at 5.0 min, then reverted to 70% A and was maintained until 10.0 min to re-establish equilibrium. The flow rate was set at 0.4 mL/min. The calibration curve for L-carnitine was established over a concentration range of 0.002–2.0 μg/mL. The limit of detection (LOD) and limit of quantification (LOQ) were 0.0043 and 0.013 μg/mL, respectively (Table S1).

**Analysis of isoflavones.** A 10 mg sample of nonfermented or fermented soybean extract powder was dissolved in DMSO, diluted to 1 mg/mL with methanol, and filtered through a 0.2 μm syringe filter (Pall). Isoflavone glucosides (daidzin, genistin, glycitin) and their aglycones (daidzein, genistein, glycitein) were analyzed using a UPLC system equipped with a BEH C_18_ column (1.7 μm, 2.1 × 100 mm, Waters, USA). Detection was performed using a QDa mass detector (Waters). The flow rate was maintained at 0.3 mL/min, with mobile phases A (acetonitrile with 0.1% formic acid) and B (distilled water with 0.1% formic acid). The gradient began at 10% A (0 min), increased to 50% at 5 min, then to 90% at 7.0 min. After 1 min at 90%, the gradient returned to 10% A and stabilized until 10 min. Daidzin, glycitin, genistin, daidzein, glycitein, and genistein, covering concentrations from 0.02 to 10 μg/mL, were used as standards. The isoflavones were expressed as μmol/kg DM.

**Analysis of β-glucosidase activity.** To extract β-glucosidase, 25 mg of either nonfermented or fermented soybean was mixed with 1 mL of distilled water and shaken at 200 rpm for 30 min. The mixture was then centrifuged at 10,000 g for 15 min at 4°C. The pellet was discarded, and the supernatant served as the crude enzyme extract. Protein content was measured using the Bradford assay with bovine serum albumin as the standard. The enzyme reaction was started by adding 150 μL of 10 mM p-NPG to a reaction mixture containing the crude enzyme in 50 mM sodium phosphate buffer (pH 6.0). The mixture was incubated at 30°C for 30 min. Afterward, 300 μL of 500 mM Na_2_CO_3_ was added, and the absorbance was measured at 405 nm with a a SpectraMax M3 microplate reader (Molecular Devices, USA). One unit of β-glucosidase activity was defined as the amount of enzyme that released 1 μmol of p-nitrophenol per minute. Activity was expressed as U/mg.

**Total phenolic (TPC) and total flavonoid content (TFC) analysis.** The Folin–Ciocalteu method was employed to measure TPC in both nonfermented and fermented soybean, as detailed in our previous study [16], using gallic acid as the standard (1–100 μg/mL). To analyze TFC, the aluminum chloride colorimetric method was applied, following the protocol in [17], with quercetin serving as the standard (1–100 μg/mL).

**Total saponin content (TSC) analysis.** The vanillin-sulfuric acid assay was employed to determine the TSC of both nonfermented and fermented soybean, using oleanolic acid as the standard [18]. In brief, the sample was mixed with 72% (v/v) sulfuric acid and 8% (w/v) vanillin at a 1:10:1 (v/v/v) ratio, then incubated at 60°C for 10 min and cooled for 5 min. The plate was read at 544 nm with a SpectraMax M3. The TSC was expressed as mg oleanolic acid equivalent per gram of dry mass (mg OAE/gDM).

### Antioxidant Capacity

**Oxygen radical absorbance capacity (ORAC) analysis.** The ORAC assay for both nonfermented and fermented soybean was performed using the method of Ou *et al*. (2001). Briefly, 10 μL of each sample was mixed with 25 μM sodium fluorescein and 25 mM AAPH in a 96-well microplate, then incubated at 37°C for 120 min. Fluorescence was measured at an excitation wavelength of 485 nm and an emission wavelength of 538 nm every 3 min. Results, calculated from the area under the fluorescence decay curve, are expressed as Trolox equivalents per gram of dry weight (mM TE/g DM).

**ABTS^+^• radical scavenging activity.** The ABTS^+^• solution was prepared by mixing 2.45 mM potassium persulfate with 7 mM ABTS in the dark at 22°C for 16 h. Afterward, it was diluted with 75 mM sodium phosphate buffer (pH 7.4) until it reached an absorbance of 0.70 ± 0.03 at 734 nm, measured using a SpectraMax M3. For the assay, 180 μL of this ABTS^+^• solution was combined with 20 μL of the sample and incubated in the dark at 22°C for 30 min. The mixture was then measured at 734 nm using a SpectraMax M3. The ABTS^+^• radical-scavenging activity was determined by comparing the results to the control.

**Ferric reducing antioxidant power (FRAP) analysis.** The FRAP assay was conducted on nonfermented and fermented soybean following our previous method [20]. The FRAP reagent was freshly prepared by combining 20 mM ferric chloride (FeCl_3_), 10 mM 2,4,6-tripyridyl-s-triazine (TPTZ) in 40 mM HCl, and 0.3 M sodium acetate buffer (pH 3.6) at 1:1:10 (v/v/v). Then, 20 μL of the sample was mixed with 180 μL of the reagent and incubated at 22°C for 30 min. The sample was measured at 593 nm using a SpectraMax M3. Results are expressed as mM Fe^2+^ per gram of dry mass (mM Fe^2+^/g DM).

**α-Glucosidase inhibitory activity.** The α-glucosidase inhibitory activity of both nonfermented and fermented soybean was assessed following a previously established method [20]. Briefly, 0.2 U/mL α-glucosidase was mixed with the sample in 50 mM potassium phosphate buffer (pH 6.8) and incubated at 37°C for 5 min. The reaction was initiated by adding 5 mM p-NPG, and the mixture was incubated at 37°C for an additional 8 min. To stop the reaction, 250 mM Na_2_CO_3_ was added (1:1, v/v), after which the sample was transferred to a 96-well plate and measured at 405 nm using a SpectraMax M3. The inhibition percentage was calculated relative to the control.

**Tyrosinase inhibition assay.** For the tyrosinase inhibition assay, 10 U/mL tyrosinase was mixed with the sample in a 50 mM potassium phosphate buffer (pH 6.5) and incubated at 37°C for 5 min. The reaction was initiated by adding 10 mM L-DOPA, followed by incubation at 37°C for 10 min. The plate was then read at 475 nm using a SpectraMax M3. The inhibition percentage was calculated relative to the control.

### Statistical Analysis

Data are presented as the mean standard deviation (SD) from three independent biological replicates (n = 3). All statistical tests were performed using SPSS (version 26.0; IBM Corp., USA). Differences between groups were examined with one-way analysis of variance (ANOVA) followed by Tukey’s multiple comparison test. A p-value less than 0.05 was regarded as statistically significant. Graphs were created with Prism 8.0 (GraphPad Software Inc., USA). Multivariate analyses, such as principal component analysis (PCA), Pearson correlation, and heatmaps, were carried out in OriginPro 2022b (OriginLab., USA).

## Results and Discussion

### Changes in the Physical Properties of Soybean by Solid-State Fermentation Using *R. oligosporus*

**Water holding (WHC) and water swelling capacity (WSC).** WHC and WSC are common terms in food science because they are among the properties that influence a food's sensory quality [21]. The WHC of soybean increased from 3.67 ± 0.06 to 5.13 ± 0.06 g/g, representing a 1.40-fold increase compared with nonfermented soybeans (Fig. 1A). WHC reached its peak after 15 days of fermentation, with no significant differences among the 10-, 15-, and 20-day fermented samples (Fig. 1A). The WSC of soybean remained unchanged until day 5 of fermentation, then increased 1.64-fold after 10 days. No significant difference in WSC was observed among fermented soybeans from day 0 to day 5 or from day 7 to day 20 (Fig. 1B). During fermentation, the microstructure changed, resulting in a looser structure and the degradation of insoluble fiber. This process creates space within the molecular structure and exposes hydrophilic groups, which improve WHC and WSC [20, 22].

**Oil absorption capacity (OAC).** OAC reflects the matrix's capacity to physically trap lipids. It increased with fermentation time (Fig. 1C). The highest OAC was observed in soybean fermented for 20 days, representing a 1.55-fold increase over nonfermented soybean. Our findings align with a prior study demonstrating that the oil-absorbing capacity (OAC) of maca increased through solid-state fermentation with *R. oligosporus* [13], similar to how the OAC of okara was enhanced using *Monascus purpureus* [20]. The improvement in OAC caused by microstructure changes may expose functional groups, which aid in physically trapping oil within the matrix [20, 22].

**Cholesterol binding capacity.** Excess cholesterol accumulation is a key risk factor for obesity, coronary heart disease, and metabolic disorders. The cholesterol-binding capacity (CBC) of soybean under gastric (pH 2.0) and intestinal (pH 7.0) conditions is illustrated in Fig. 1D−E. No significant differences in CBC were observed between nonfermented and fermented soybeans after 3, 5, 7, 10, or 15 days at pH 2.0 (Fig. 1D). However, CBC increased by 1.43- and 1.49-fold after 15 and 20 days of fermentation, respectively, with a significant increase observed after 20 days. At pH 7.0, CBC values increased from 2.27 ± 0.15 mg/g in nonfermented to 2.71 ± 0.14 mg/g after 15 days of fermentation, although the difference was not statistically significant (Fig. 1E).

### Changes in the Phytochemical Components of Soybean by Solid-State Fermentation Using *R. oligosporus*

**Ergosterol content.** Ergosterol is a vital sterol present in the cell membranes of most fungi and is used to monitor fungal growth during solid-state fermentation. Additionally, it is a bioactive compound that promotes lipid peroxidation and exhibits antioxidant, anticancer, and cholesterol-lowering properties [23, 24]. Fig. 2A illustrates the variations in ergosterol content during soybean fermentation with *R. oligosporus*. Ergosterol was not detected in nonfermented soybean but increased to 13.5 ± 0.12 mg/g DM after 20 days of fermentation. The highest ergosterol concentration was recorded on day 20 of fermentation (Fig. 2A). These results are consistent with previous studies showing ergosterol levels rising from 16.2 ± 0.03 μg/g to 488.5 ± 1.3 μg/g after 10 days of fermenting wild simulated ginseng leaves [15], and from 40.6 ± 1.1 μg/g in nonfermented maca to 904.3 ± 3.3 μg/g following 10 days of maca fermentation [13]. A rise in ergosterol content during solid-state fermentation clearly indicates vigorous mycelial growth and efficient adaptation of *R. oligosporus* to soybean.

**L-carnitine content.** Fig. 2B shows the L-carnitine levels in nonfermented and fermented soybean. Initially, soybean contained 0.66 ± 0.15 mg/kg DM. There was no significant change until day 5 of fermentation. By day 7, the content increased dramatically to 11.22 ± 1.17 mg/kg DM, a 17.0-fold increase over nonfermented soybean. After 7 days, L-carnitine surged to 77.8 ± 6.77 mg/kg DM on day 10 and further increased to 100.77 ± 2.19 mg/kg DM on day 15. The peak was reached on day 20, with 161.21 ± 3.78 mg/kg DM, representing a 244.26-fold increase over nonfermented soybean (Fig. S1). A similar L-carnitine accumulation has been reported in other plant-based substrates fermented with *R. oligosporus* [13, 15]. In maca, L-carnitine was not detected during the first two days, appeared on day 3, and increased markedly by day 15 to 157.32 ± 5.57 mg/kg DM [13]. Likewise, L-carnitine accumulated in wild-simulated ginseng leaves from undetectable levels to 165.1 ± 1.8 μg/g after 10 days of fermentation, while the highest L-carnitine content in fermented quinoa was observed on day 3. [25]. The accumulation of L-carnitine during solid-state fermentation (SSF) of soybean by *Rhizopus oligosporus* is likely attributable to the activation of the fungal L-carnitine biosynthetic pathway together with the increased availability of precursor amino acids. During SSF, *R. oligosporus* secretes a range of extracellular hydrolases, including proteases, endo- and exo-glucanases, β-glucosidases, and esterases, which degrade plant cell wall components and proteins, thereby releasing nutrients required for fungal growth and metabolism [9, 26, 27]. Among these enzymes, proteases play a particularly important role by liberating amino acids such as lysine and methionine, the two essential precursors for L-carnitine biosynthesis. Over 40 U/g protease was produced during 60 h of SSF by *R. oligosporus*, resulting in increased concentrations of soluble proteins and low-molecular-weight peptides [9]. Consistent with these observations, Ryu *et al*. (2024) reported significant increases in methionine (0.48–1.32 mg/g) and leucine (3.33–5.78 mg/g) following fermentation of maca, suggesting that fungal fermentation enhances amino acid availability. In fungi, L-carnitine is synthesized from L-lysine and L-methionine through a conserved four-step biosynthetic pathway that was first characterized in *Neurospora crassa*. In this pathway, methyl groups donated by S-adenosyl-L-methionine, which is generated from methionine, are transferred to lysine to form Nε-trimethyllysine (TML). TML is subsequently converted to L-carnitine through sequential hydroxylation to β-hydroxy-TML, cleavage to γ-trimethylaminobutyraldehyde, oxidation to γ-butyrobetaine, and a final hydroxylation catalyzed by γ-butyrobetaine hydroxylase [28]. Therefore, the marked accumulation of L-carnitine observed during soybean fermentation is likely the result of both enhanced precursor availabilities through proteolysis and the active fungal biosynthetic machinery. Additionally, the parallel increase in L-carnitine and ergosterol suggests that L-carnitine accumulation is associated with the progression of fungal growth and metabolic activity in the system. L-carnitine synthesis in the brain, kidneys, and liver accounts for only 25%, with the remaining 75% obtained through diet. The main dietary source of L-carnitine is red meat, such as pork and beef, while plant-based foods contain only small amounts, making it difficult to obtain sufficient amounts from a plant-based diet [29]. Since soybeans are a key component of such diets, L-carnitine-enriched fermented soybean is a suitable ingredient for plant-based foods.

**Total phenolic, flavonoid, and saponin contents.** Polyphenols, flavonoids, and saponins are important secondary metabolites in soybeans, contributing to antioxidant and other health-related properties [30, 31]. Fermentation with *R. oligosporus* is known to modulate their levels and bioavailability [15, 25, 32]. Accordingly, this study evaluated the changes in total phenolic content (TPC), total flavonoid content (TFC), and total saponin content (TSC) of soybean during solid-state fermentation with *R. oligosporus*, and the results are shown in Fig. 2C−2E. TPC reached its maximum on day 15 with 25.01 ± 0.65 mg GAE/g DM, which is 3.13-fold higher than in nonfermented soybean. Afterward, it slightly declined on day 20 (Fig. 2C). The TFC of soybean peaked on day 10, showing a 3.84-fold increase over nonfermented soybean. Following this peak, TFC decreased on days 15 and 20. However, no significant differences in TFC were observed among days 5, 15, and 20 of fermentation (Fig. 2D). The TSC of soybean increased with fermentation time. It peaked on day 20 at 232.94 ± 0.57 mg OAE/g DM, which is 2.52-fold higher than in nonfermented soybean (Fig. 2E). Lim *et al*. (2023) observed that fermenting wild ginseng leaves with *R. oligosporus* under solid-state conditions resulted in a peak TSC of 82.1 ± 9.1 mg/g after 5 days, marking a 1.43-fold increase. The TPC was highest on day 3 of fermentation. Ryu *et al*. (2024) fermented maca with *R. oligosporus* and observed that both TPC and TFC peaked on day 5, increasing by 1.18- and 1.88-fold, respectively. Furthermore, TSC peaked on day 7, showing a 2.20-fold increase. Zhang *et al*. (2022) showed that during the solid-state fermentation with *R. oligosporus* RT3, enzymes such as α-amylase, cellulase, endoglucanase, exoglucanase, β-glucosidase, esterase, and protease were produced, with their levels increasing over fermentation time [9]. These enzymes degrade the cell wall matrix and release free and bound phenolic compounds, thereby increasing TPC, TFC, and TSC during fermentation.

**β-Glucosidase activity, isoflavone glycosides, and isoflavone aglycones.** Soybeans contain two forms of isoflavones: glucosides and aglycones. Aglycones are absorbed faster and are more abundant in humans than glucosides, making them more effective at preventing chronic diseases [33]. The enzyme β-glucosidase is essential because it hydrolyzes β-glycosidic bonds, converting glucosides into aglycones. Its activity in fermented soybean was measured, as shown in Fig. 3A. No activity was observed in nonfermented soybean. However, it increased to 10.89 ± 0.10 U/mg protein by day 15 of fermentation and then slightly declined to 8.95 ± 0.20 U/mg protein by day 20. A similar pattern was observed in fermented soybean and wild-simulated ginseng leaves fermented with *R. oligosporus*, where β-glucosidase activity was produced and increased during solid-state fermentation [9, 15]. Additionally, fermented soybeans produced more β-glucosidase than fermented wild-simulated ginseng leaves [15]. Furthermore, β-glucosidase also participates in degrading saponin glucosides into saponin aglycones, resulting in increased total saponin content [34].

During solid-state fermentation with *R. oligosporus*, the isoflavone glucosides daidzin, genistin, and glycitin decreased progressively, whereas their corresponding aglycones showed compound-specific, time-dependent changes (Fig. 3B−3D). Initially, daidzin was measured at 289.32 ± 16.19 μmol/kg DM in unfermented soybean, remained at that level after 3 days of fermentation, and became undetectable after 15 days. Conversely, daidzein, the aglycone of daidzin, increased from 37.26 ± 19.16 μmol/kg DM to 691.06 ± 36.98 μmol/kg DM after 15 days, representing an 18.55-fold rise, before slightly decreasing to 597.88 ± 44.72 μmol/kg DM after 20 days (Fig. 3B). Genistin was found at 1681.57 ± 21.89 μmol/kg DM in nonfermented soybean, with a slight increase after 3 days of fermentation. However, there was no significant difference in genistin content among nonfermented, 3-day, and 5-day fermented soybeans. Genistin levels declined with ongoing fermentation and became undetectable after 20 days. Conversely, genistein, the aglycone of genistin, was not present in nonfermented soybeans and reached a peak of 1212.44 ± 43.32 μmol/kg DM after 10 days of fermentation. Subsequently, its level dropped to 149.99 ± 34.34 μmol/kg DM by day 20 (Fig. 3C). Glycitin levels in soybean rose by 1.19-fold after 3 days of fermentation, then dropped sharply by 17.34-fold after 15 days, and became undetectable after 20 days of fermentation. Conversely, glycitein, which is the aglycone form of glycitin, was present in nonfermented soybean at 82.96 ± 2.47 μmol/kg DM. Its level then increased to 238.70 ± 4.19 μmol/kg DM, representing a 2.88-fold increase compared with nonfermented soybean (Fig. 3D). Fig. 3E also shows the total isoflavone glucosides (daidzin, genistin, glycitin), the total isoflavone aglycones (daidzein, genistein, glycitein), and the combined six isoflavones measured in this study. Although the difference in isoﬂavone glucosides of fermented soybean between days 3 and 5 was not statistically significant, levels of isoﬂavone aglycones and overall isoflavones increased by 7.25 and 1.33 times, respectively. The total isoflavone glucosides in soybean decreased from 3118.38 ± 118.20 μmol/kg at day 3 to 15.16 ± 17.61 μmol/kg at day 5, a 99.51% reduction. Total isoflavones also went down from 3989.5 ± 154.70 μmol/kg DM at day 3 to 1001.73 ± 101.58 μmol/kg DM, a decrease of 74.89%. In contrast, the total isoflavone aglycones peaked at 1892.75 ± 61.87 μmol/kg DM on day 10 before decreasing to 986.58 ± 83.97 μmol/kg DM by day 20 (Fig. 3E). Our results were consistent with previous studies using *R. oligosporus* to ferment soybean and black soybean, in which isoflavone glycosides were effectively converted to their corresponding aglycones [9, 35]. Zhang *et al*. (2022) reported that after 60 h of fermentation with *R. oligosporus* RT-3, isoflavone glucosides decreased: daidzin by 3.41-fold, genistin by 1.96-fold, and glycitin by 1.50-fold. Meanwhile, the levels of daidzein, genistein, and glycitein increased by 4.71-, 3.07-, and 3.97-fold, respectively. Isoflavones in soybean are classified into four forms: aglycones, β-glucoside, acetyl glucoside, and malonyl glucoside [36]. The observed increase in isoflavone aglycones and total isoflavones after three days of fermentation is attributed to enzymes such as α-amylase, cellulase, endoglucanase, exoglucanase, β-glucosidase, esterase, and protease, which are produced during fermentation. These enzymes degrade the cell wall matrix, releasing both free and bound isoflavones [9]. The decline in isoflavones during fermentation may be due to oxidation, polymerization, or further transformation into low-molecular-weight metabolites [37, 38].

### Changes in the Functional Properties of Soybean by Solid-State Fermentation Using *R. oligosporus*

**Antioxidant activity.** The antioxidant activity of soybean during solid-state fermentation with *R. oligosporus* was assessed using ORAC, FRAP, and ABTS assays (Fig. 4A−4C). ORAC values increased steadily from 5.06 ± 0.05 to 9.75 ± 0.09 mM TE/g DM, peaking at day 20, which was 1.93-fold higher than that of nonfermented soybean. No significant differences in ORAC were observed between 5 and 7 days or between 15 and 20 days (Fig. 4A). FRAP showed a similar upward trend until day 15, reaching 213.46 ± 5.95 mM Fe^2+^/g DM, and then declined slightly on day 20 (Fig. 4B). The ABTS radical-scavenging activity was evaluated using the SC_50_ value, where a lower SC_50_ reflects a stronger antioxidant effect. The SC_50_ value of soybean dropped from 1.65 ± 0.04 mg/mL in nonfermented soybean to 1.10 mg/mL after 20 days of fermentation, reflecting a 1.5-fold increase in antioxidant capacity (Fig. 4C). The increased antioxidant activity during fermentation may be associated with L-carnitine accumulation and higher levels of ergosterol, TPC, TFC, TSC, and isoflavone aglycones [39, 40].

**α-Glucosidase inhibitory activity.** α-Glucosidase is a membrane-bound enzyme in the small-intestinal epithelium that breaks down disaccharides into monosaccharides for energy. However, excessive activity of this enzyme can cause complications, especially in patients with hyperglycemia. Inhibiting α-glucosidase slows carbohydrate absorption, lowers post-meal blood glucose levels, and is therefore a vital therapeutic target in diabetes. Isoflavone aglycones exhibit greater α-glucosidase inhibitory activity than acarbose, a common medication for type 2 diabetes [41]. Therefore, the effects of fermented soybean with *R. oligosporus* on α-glucosidase activity were investigated, and the results are shown in Fig. 5A as IC_50_ values. A lower IC_50_ indicates stronger inhibition. Nonfermented soybean had an IC_50_ of 96.74 ± 2.48 μg/mL. After 3 days of fermentation, the IC_50_ decreased to 56.52 ± 0.73 μg/mL, a 1.71-fold increase in α-glucosidase inhibitory activity compared with nonfermented soybean. The IC_50_ reached its lowest value on day 7 of fermentation at 41.83 ± 0.44 μg/mL, a 2.31-fold increase over the nonfermented soybean. Later, the IC_50_ value rose during fermentation, reaching 61.92 ± 0.48 μg/mL by day 20. Choi *et al*. (2010) found that isoflavone aglycones, such as daidzein and genistein, have stronger inhibitory activity than their glucosides, with IC_50_ values of 0.85 mM and 0.15 mM, respectively. In comparison, daidzin and genistin had IC_50_ values exceeding 30 mM [41]. During solid-state fermentation with *R. oligosporus*, soybean isoflavone glucosides were transformed into their isoflavone aglycones (Fig. 3B−3D), enhancing α-glucosidase inhibitory activity.

**Tyrosinase inhibitory activity.** Tyrosinase is a crucial enzyme that controls melanogenesis, the process of melanin production in the cells of the human eye, skin, and hair. Increased melanin can lead to skin conditions such as age spots, freckles, melasma, and malignant melanoma. Several studies have indicated that traditional Korean fermented soybean foods, including Cheonggukjang and Doenjang, have notable inhibitory effects [35, 36]. Therefore, the inhibitory effects of fermented soybean with *R. oligosporus* against tyrosinase activity were investigated. The results are presented in Fig. 5B. Nonfermented soybean exhibited a tyrosinase inhibitory activity with an IC_50_ of 1.78 ± 0.07 mg/mL. The IC_50_ value decreased as fermentation progressed, reaching its lowest point of 1.19 ± 0.03 mg/mL after 10 days, indicating a 1.50-fold increase in tyrosinase inhibitory activity compared with nonfermented soybean. Subsequently, the IC_50_ value gradually increased again up to 20 days of fermentation.

**Correlation analysis.** Pearson correlation analysis was used to assess the relationship between bioactive compounds and the functional properties of fermented soybean over 0-20 days, with results displayed as a heatmap in Fig. 5C. Antioxidant activity measured by FRAP and ORAC assays showed strong positive correlations with glycitein (r ≥ 0.93), daidzein (r ≥ 0.96), ergosterol (r ≥ 0.88), and L-carnitine (r ≥ 0.8). Additionally, it showed positive correlations with OAC (r ≥ 0.9), WHC (r ≥ 0.95), TPC (r ≥ 0.94), and TSC (r ≥ 0.95). Conversely, it was significantly negatively correlated with daidzin (r ≤ -0.84), genistin (r ≤ -0.84), and glycitin (r ≤ -0.84), as shown in Fig. 5C. The ABTS radical-scavenging activity, measured by the SC_50_ value, exhibited strong positive correlations with daidzin (r = 0.98), genistin (r = 0.96), and glycitin (r = 0.92). Since a lower SC_50_ indicates higher antioxidant activity, these positive correlations suggest that increased levels of glucosides are associated with decreased ABTS scavenging activity. Conversely, negative correlations were observed with L-carnitine (r = -0.91), ergosterol (r = -0.964), β-glucosidase (r = -0.93), daidzein (r = -0.90), glycitein (r = -0.88), WHC (r = -0.91), and OAC (r = -0.96), indicating that these compounds are linked to improved ABTS radical-scavenging activity in fermented soybeans. The α-glucosidase inhibitory activity, expressed as an IC_50_, showed strong negative correlations with genistein (r = -0.78) and TFC (r = -0.76) (Fig. 5C), implying that higher TFC and genistein levels enhance α-glucosidase inhibition. Meanwhile, tyrosinase inhibitory activity, also expressed as an IC_50_, was positively correlated with daidzin (r = 0.92) but negatively with TFC (r = -0.86), β-glucosidase (r = -0.89), genistein (r = -0.82), daidzein (r = -0.81), and WHC (r = -0.84), suggesting that TFC, β-glucosidase, genistein, and daidzein promote tyrosinase inhibition, while high levels of daidzein may reduce it. Furthermore, PCA was used to examine the correlations among fermentation time, phytochemical compounds, and functional properties, and the results are shown in Fig. 5D. PC1 explained 77.64% of the variance, and PC2 explained 13.58%, accounting for 91.22% of the total variability. The variance of PC1 was determined by TPC, TSC, FRAP, ORAC, ergosterol, β-glucosidase, daidzein, glycitein, WHC, OAC, WSC, and CBC at pH 2.0, with correlation factors (r≥0.20) on the right side of the PCA plot, whereas ABTS SC_50_, daidzin, glycitin, and genistin, with correlation factors (r ≤ -0.23) were located on the left side of the PCA plot. The variance of PC2 was determined by L-carnitine, α-glucosidase inhibitory activity, tyrosinase inhibitory activity, and CBC at pH 2.0 with correlation factors (r ≥ 0.246) on the right side of the PCA plot, whereas TFC and genistein showed strong negative correlation factors of r = -0.35 and r = 0.50, respectively (Fig. 5). The PCA outcomes demonstrated that fermented soybean with *R. oligosporus* at various fermentation times occupied distinct positions in the PCA space, indicating clear differences in phytochemical content, biological activity, and physical properties across different fermentation stages.

## Conclusion

In summary, soybeans enriched with L-carnitine and ergosterol were produced via solid-state fermentation with *R. oligosporus*. This process enhanced the soybeans' physical properties, including water-holding, water-swelling, oil-absorption, and cholesterol-binding capacities at pH 2.0. However, no significant change in cholesterol binding was observed at pH 7.0. Fermentation significantly increased TPC, TFC, and TSC by 3.13-, 3.84-, and 2.52-fold, respectively. Additionally, isoflavone glucosides progressively decreased during fermentation, accompanied by compound-specific changes in their corresponding aglycones. Daidzein increased from to 37.26 ± 19.16 to 691.06 ± 36.98 μmol/kg DM after 15 days, genistein increased from undetectable levels to 1212.44 ± 43.32 μmol/kg DM after 10 days before declining thereafter, and glycitein increased from 82.96 ± 2.47 to 238.70 ± 4.19 μmol/kg DM. Fermented soybean also exhibited higher antioxidant activity as measured by ORAC, ABTS, and FRAP assays, and showed inhibitory effects against α-glucosidase and mushroom tyrosinase. The increased levels of TPC, TFC, TSC, and L-carnitine, along with the conversion of isoflavone glucosides to aglycones, enhanced its functional properties. Fermented soybean could serve as a potential functional food ingredient, prompting additional research on its microbiological safety, bioaccessibility, and biological effectiveness.

## Supplemental Materials

Supplementary data for this paper are available on-line only at http://jmb.or.kr.



## Figures and Tables

**Fig. 1:**
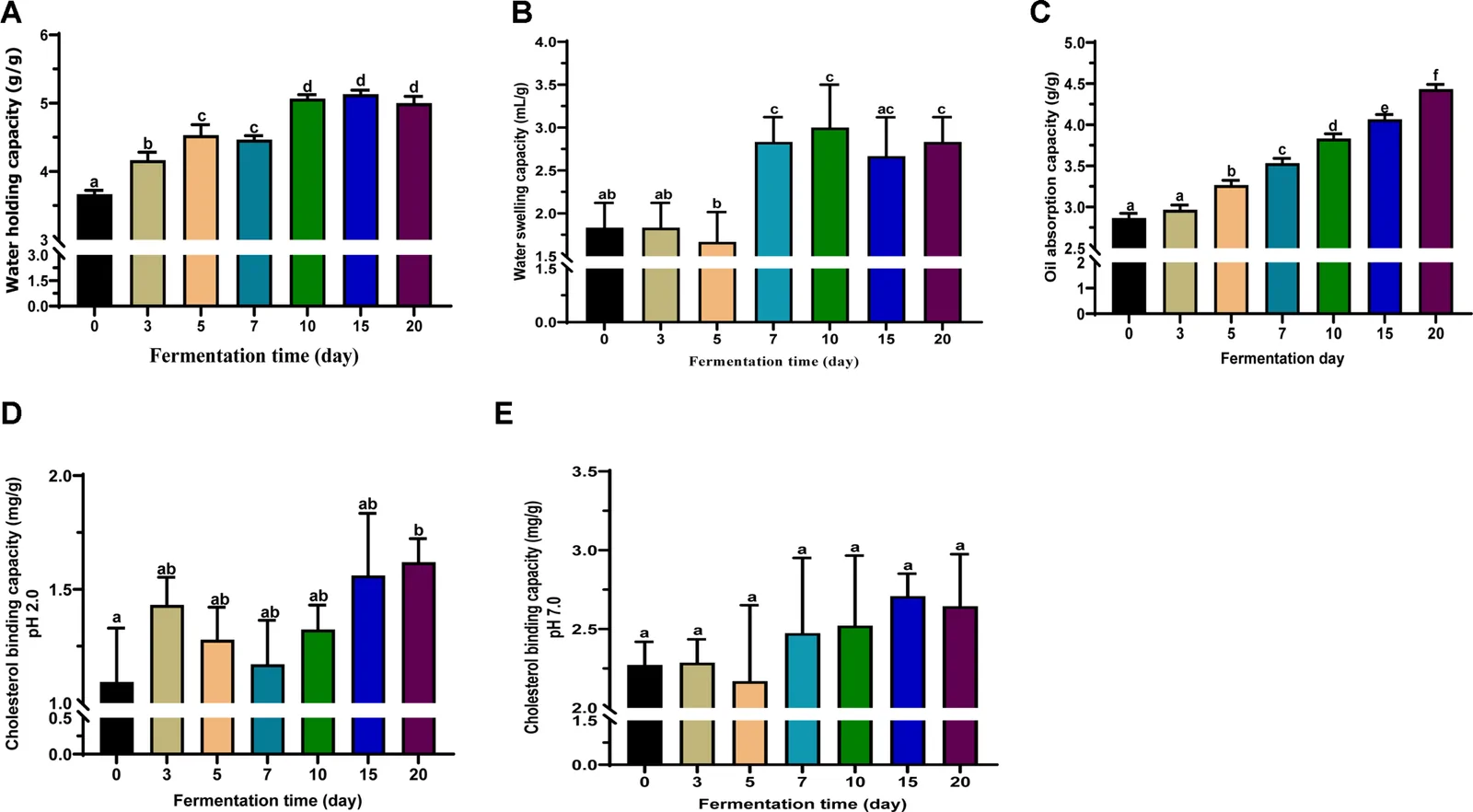
Physical properties of soybean fermented with *Rhizopus oligosporus*. (**A**) Water-holding capacity. (**B**) Water-swelling capacity. (**C**) Oil absorption capacity. (**D**) Cholesterol-binding capacity at pH 2.0. (**E**) Cholesterol-binding capacity at pH 7.0. Data are presented as the mean standard deviation (SD) of three biological replicates (*n* = 3). Different letters indicate significant differences (*p* < 0.05) according to Tukey’s multiple-comparison test.

**Fig. 2:**
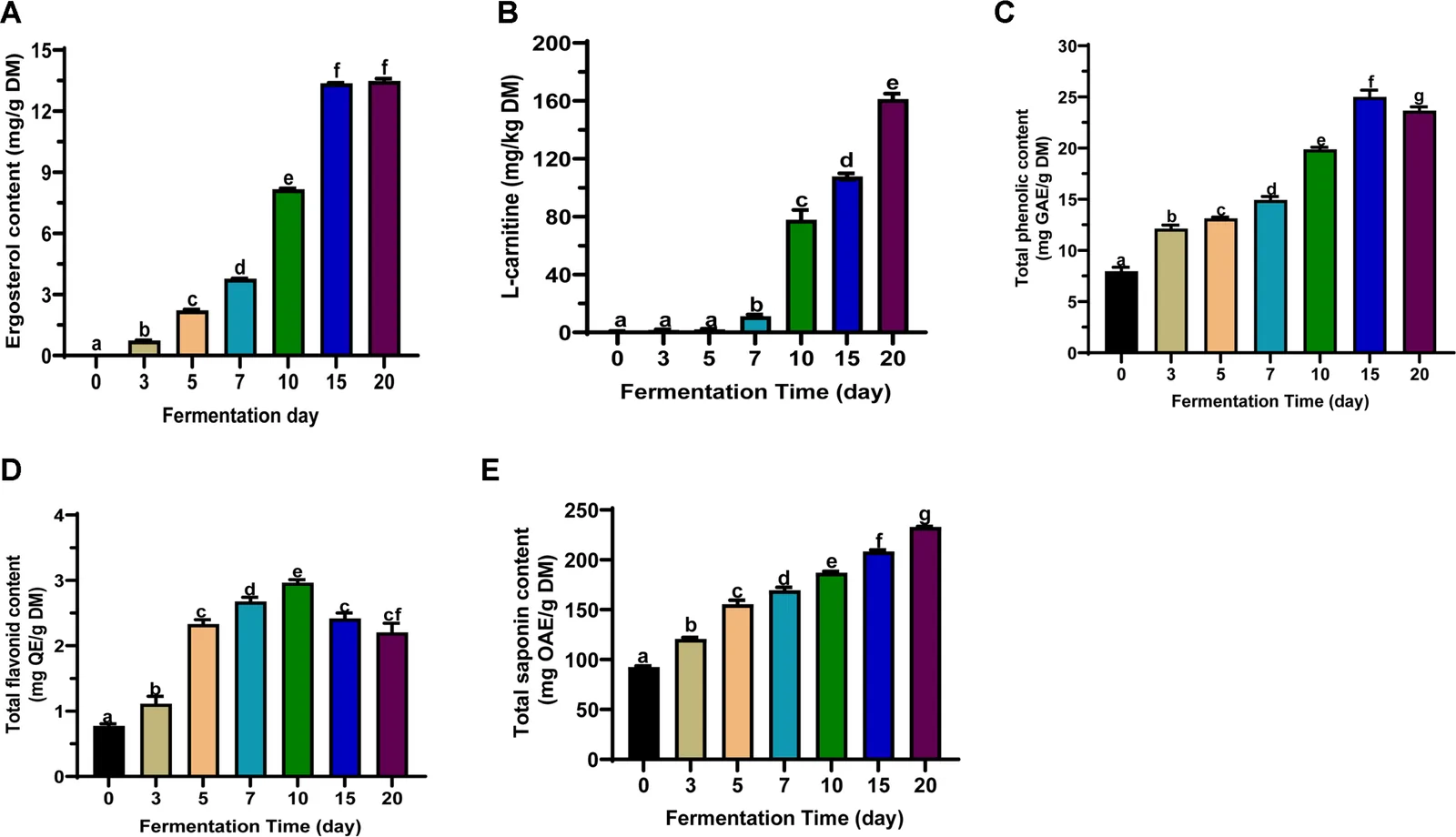
Effects of solid-state fermentation with *R. oligosporus* on ergosterol (**A**) L-carnitine (**B**) total phenolic content (**C**) total flavonoid content (**D**) and total saponin content (**E**) in soybean. Data are presented as the mean standard deviation (SD) of three biological replicates (*n* = 3). Different letters indicate significant differences (*p* < 0.05) according to Tukey’s multiple-comparison test.

**Fig. 3:**
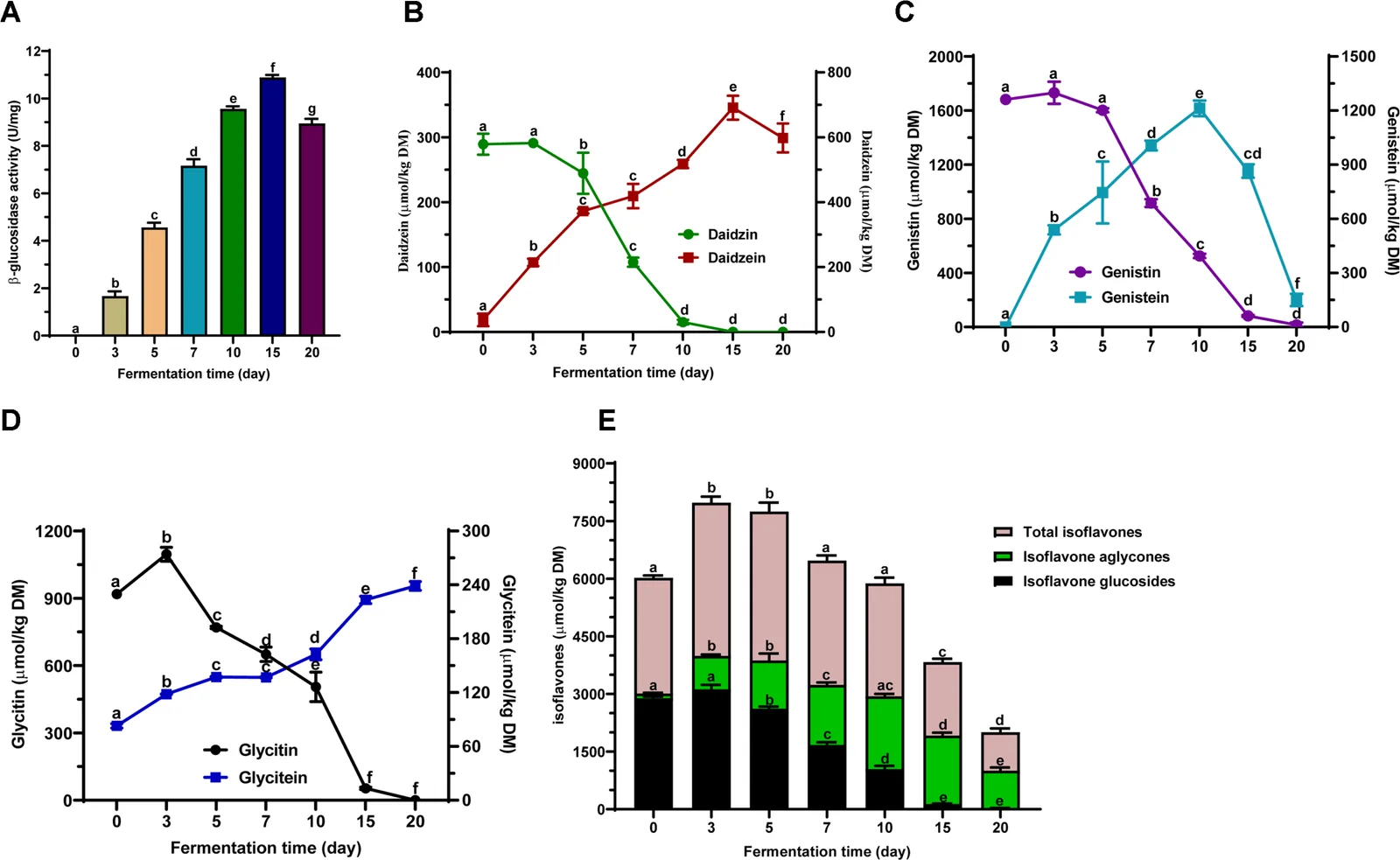
Effects of solid-state fermentation with *R. oligosporus* on β-glucosidase activity (A) isoflavone contents of daidzin and daidzein (B) genistin and genistein (C) glycitin and glycitein (D) and total isoflavone glycosides (daidzin, genistin, glycitin), total isoflavone aglycones (daidzein, genistein, glycitein), and total six isoflavones (E) on soybean. Data are presented as the mean standard deviation (SD) of three biological replicates (*n* = 3). Different letters indicate significant differences (*p* < 0.05) according to Tukey’s multiple-comparison test.

**Fig. 4:**
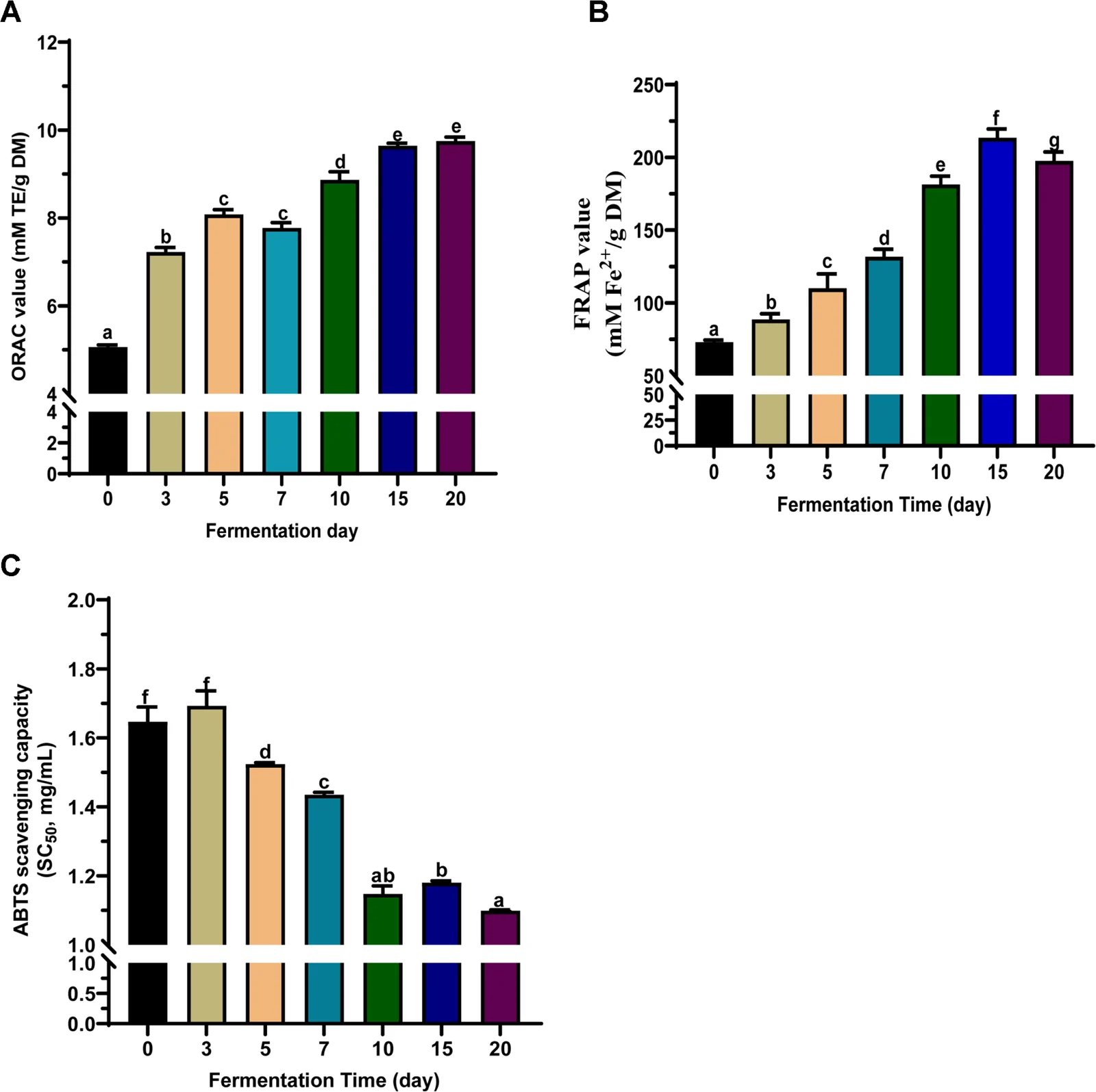
Antioxidant activities of soybean fermented with *Rhizopus oligosporus*, determined by oxygen radical absorbance capacity (ORAC) (A) ferric reducing antioxidant power (FRAP) (B) and ABTS radical-scavenging activity (C). Data are presented as the mean standard deviation (SD) of three biological replicates (*n* = 3). Different letters indicate significant differences (*p* < 0.05) according to Tukey’s multiple-comparison test.

**Fig. 5:**
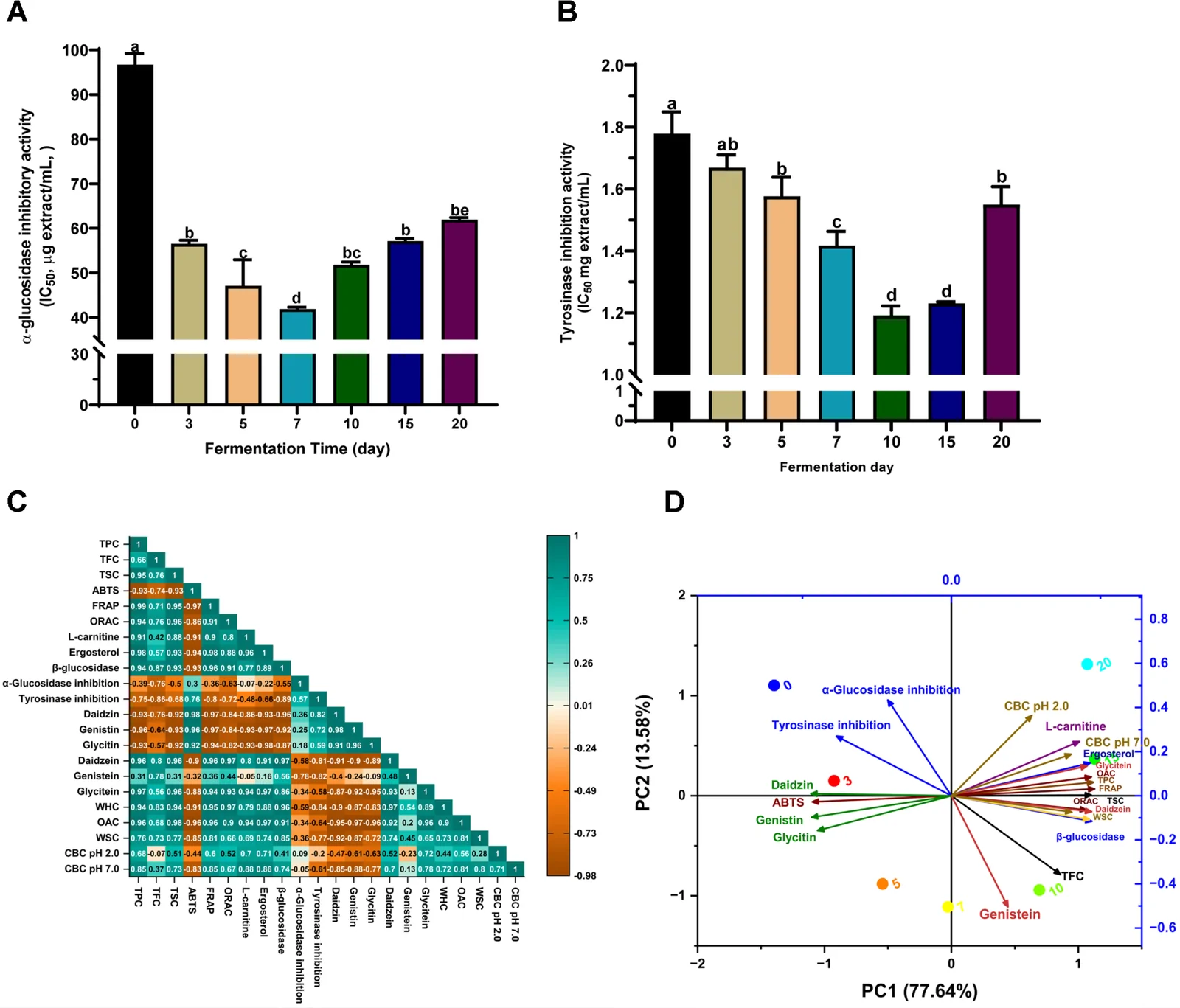
α-Glucosidase inhibitory activity (A) tyrosinase inhibitory activity (B) heatmap of Pearson correlation analysis (C) and principal component analysis (PCA) score plot (D) of soybean fermented with *R. oligosporus*. Data are presented as the mean standard deviation (SD) of three biological replicates (*n* = 3). Different letters indicate significant differences (*p* < 0.05) according to Tukey’s multiple-comparison test. The Pearson correlation coefficient (*r*) ranges from −1 (perfect negative correlation) to +1 (perfect positive correlation), as indicated by the color scale.
