# Supplementary material for: Solid-State Fermentation Drives L-Carnitine Accumulation and Isoflavone Bioconversion with Functional Enhancement in Soybean
Source: J Microbiol Biotechnol. 2026 Aug 26;36:e2606042. doi: 10.4014/jmb.2606.06042 (PMC13630530; doi:10.4014/jmb.2606.06042)
Supplement: Supplementary file 1 [file jmb-36-e2606042-supple.pdf]

J. Microbiol. Biotechnol. 2026. 36: e2606042
https://doi.org/10.4014/jmb.2606.06042

Supplementary Table and Figure

Solid-State Fermentation Drives L-Carnitine Accumulation and Isoflavone Bioconversion with Functional Enhancement in Soybean

Tri Nhut Pham[1,2], Hayeong Kim[3*], Ghahyun Jeffrey Kim[4], Soyoung Park[5], Taeyoung Um[6], Soochul Park[3,7], Doman Kim[1,3,*]

[1]Graduate School of International Agricultural Technology, Seoul National University, Gangwon-do, 25354, Republic of Korea

[2] Institute of Applied Technology and Sustainable Development, Nguyen Tat Thanh University, Ho Chi Minh City, 700000, Vietnam

[3] Institute of Food Industrialization, Institutes of Green Bioscience and Technology, Seoul National University, Gangwon-do, 25354, Republic of Korea

[4]Department of Clinical Pharmacy Practice, School of Pharmacy &Pharmaceutical Sciences, UC Irvine, CA, 92617, USA

[5]Metabolic Engineering Division, National Institute of Agricultural Sciences, Rural Development Administration, Jeonju, 54874, Republic of Korea

[6]Department of Plant Science, Kangwon National University, Gangneung, 25457, Republic of Korea

[7]Mongta Incorporation, Digital-ro, Guro-gu, Seoul, 08380, Republic of Korea.

***Corresponding author:** Doman Kim; kimdm@snu.ac.kr

***Co-corresponding author:** Hayeong Kim; hara2910@snu.ac.kr

Table S1. Calibration equation, linearity, limit of detection (LOD), and limit of quantification (LOQ) for L-carnitine analysis

**Calibration**		0.002 – 2 µg/mL
**Equation**		R^2
1	Y= 20200000X + 1540000	0.99
2	Y= 20100000X + 1630000	0.99
3	Y= 20400000X + 1560000	0.99
LOD (µg/mL)		0.0043
LOQ (µg/mL)		0.013

A)

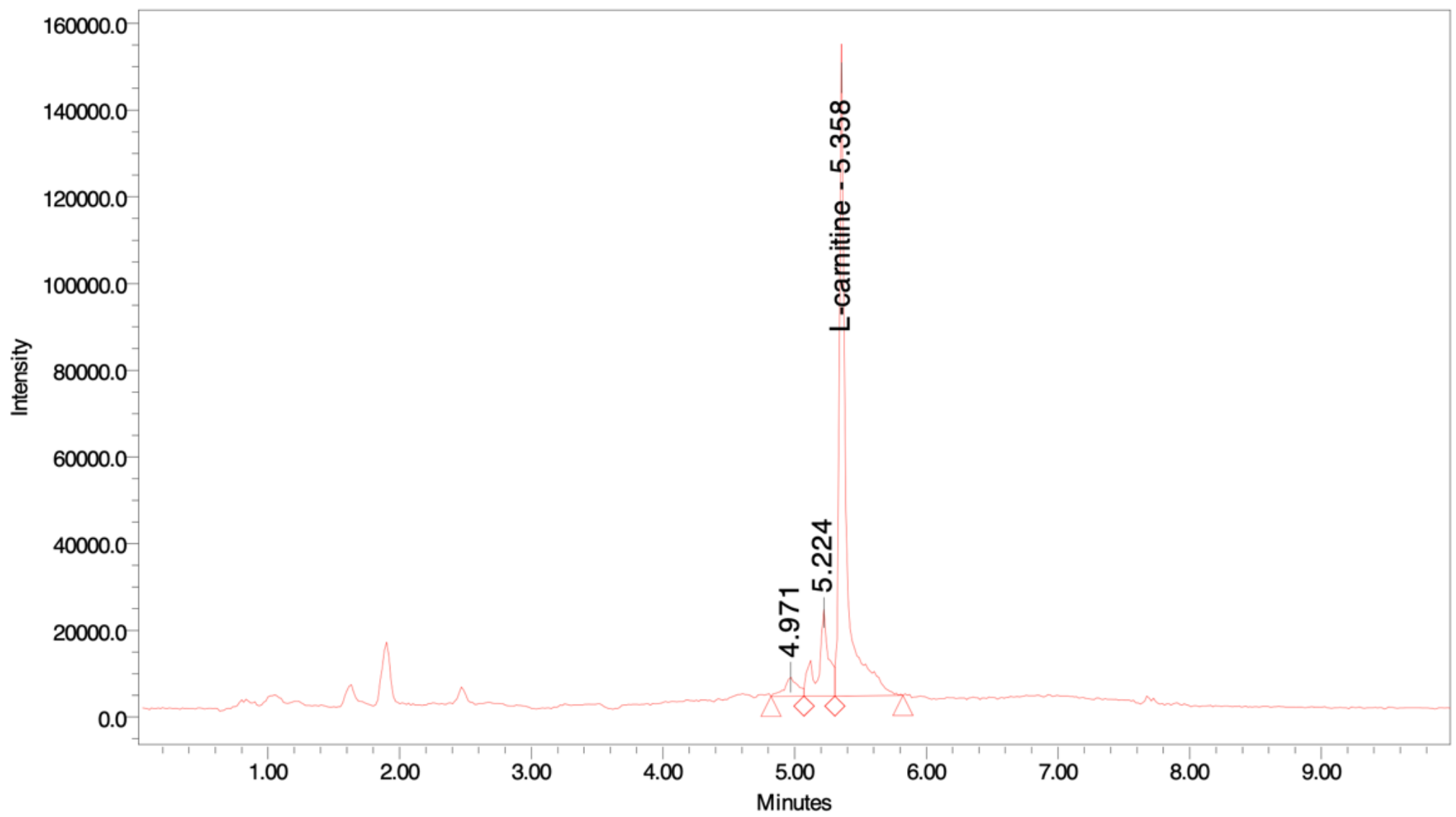

B)

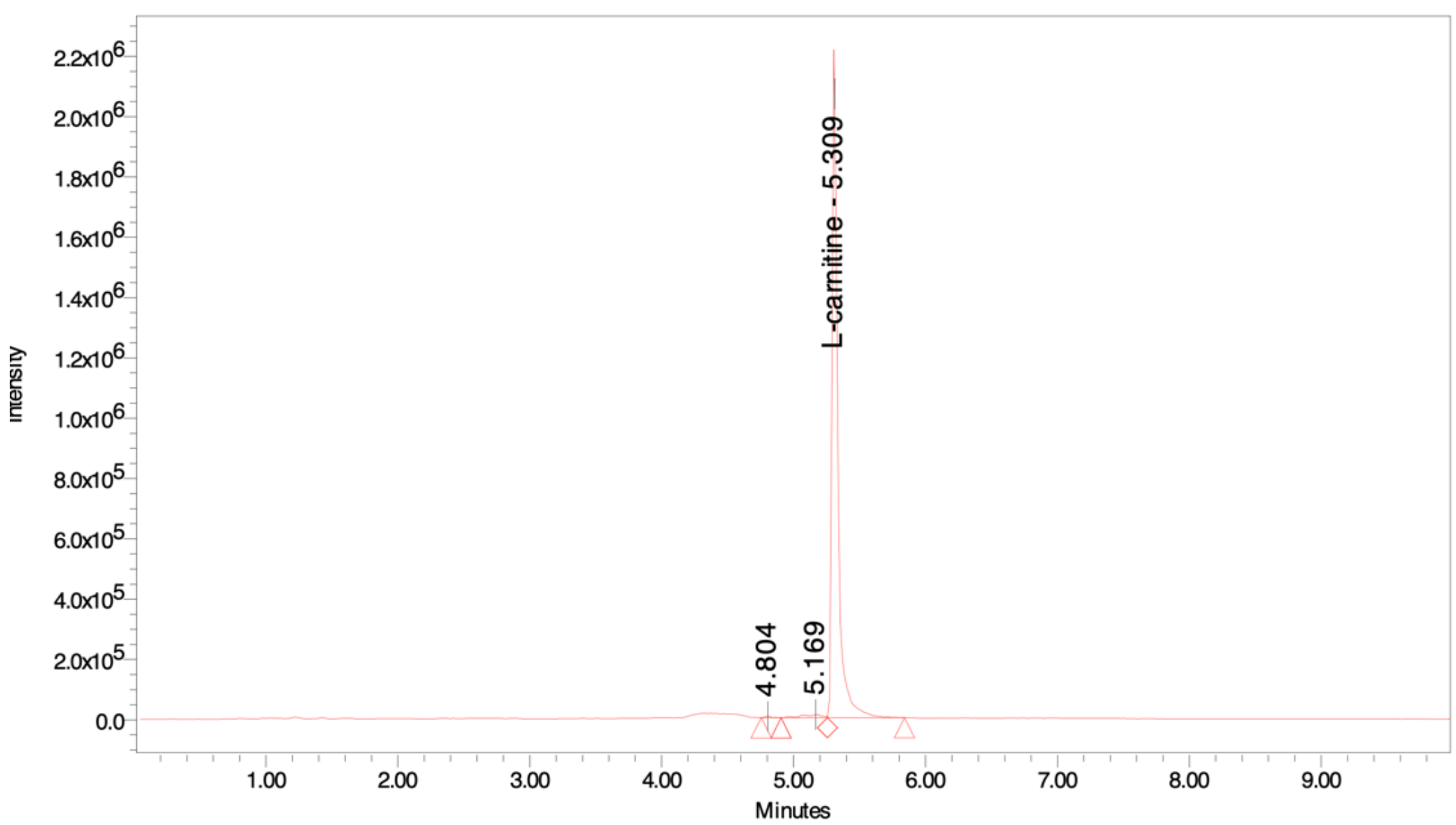

Fig. S1. Chromatogram analysis of L-carnitine of nonfermented (A) and fermented soybean at 120 h (B) using UPLC-MS with a BEH HILIC column, using acetonitrile with 0.1% (v/v) formic acid (A) and 15 mM ammonium formate with 0.1% (v/v) formic acid (B) as the mobile phase.
